# Integrative Genome-Based Survey of the SARS-CoV-2 Omicron XBB.1.16 Variant

**DOI:** 10.3390/ijms241713573

**Published:** 2023-09-01

**Authors:** Fabio Scarpa, Ilenia Azzena, Alessandra Ciccozzi, Marta Giovanetti, Chiara Locci, Marco Casu, Pier Luigi Fiori, Alessandra Borsetti, Eleonora Cella, Miriana Quaranta, Stefano Pascarella, Daria Sanna, Massimo Ciccozzi

**Affiliations:** 1 Department of Biomedical Sciences, University of Sassari, 07100 Sassari, Italy; 2 Department of Veterinary Medicine, University of Sassari, 07100 Sassari, Italy; 3 Unit of Medical Statistics and Molecular Epidemiology, Università Campus Bio-Medico di Roma, 00128 Rome, Italy; 4 Instituto Rene Rachou Fundação Oswaldo Cruz, Belo Horizonte 30190-009, MG, Brazil; 5 Sciences and Technologies for Sustainable Development and One Health, Università Campus Bio-Medico di Roma, 00128 Rome, Italy; 6 Azienza Ospedaliera Universitaria (AOU) Sassari, 07100 Sassari, Italy; 7 National HIV/AIDS Research Center (CNAIDS), National Institute of Health, 00161 Rome, Italy; 8 Burnett School of Biomedical Sciences, University of Central Florida, Orlando, FL 32816, USA; 9 Department of Biochemical Sciences “A. Rossi Fanelli”, Sapienza Università di Roma, 00185 Rome, Italy

**Keywords:** molecular evolution, epidemiology, genetics, XBB.1.16, phylodynamics, genome-based survey

## Abstract

The XBB.1.16 SARS-CoV-2 variant, also known as *Arcturus*, is a recent descendant lineage of the recombinant XBB (nicknamed *Gryphon*). Compared to its direct progenitor, XBB.1, XBB.1.16 carries additional spike mutations in key antigenic sites, potentially conferring an ability to evade the immune response compared to other circulating lineages. In this context, we conducted a comprehensive genome-based survey to gain a detailed understanding of the evolution and potential dangers of the XBB.1.16 variant, which became dominant in late June. Genetic data indicates that the XBB.1.16 variant exhibits an evolutionary background with limited diversification, unlike dangerous lineages known for rapid changes. The evolutionary rate of XBB.1.16, which amounts to 3.95 × 10^−4^ subs/site/year, is slightly slower than that of its direct progenitors, XBB and XBB.1.5, which have been circulating for several months. A Bayesian Skyline Plot reconstruction suggests that the peak of genetic variability was reached in early May 2023, and currently, it is in a plateau phase with a viral population size similar to the levels observed in early March. Structural analyses indicate that, overall, the XBB.1.16 variant does not possess structural characteristics markedly different from those of the parent lineages, and the theoretical affinity for ACE2 does not seem to change among the compared variants. In conclusion, the genetic and structural analyses of SARS-CoV-2 XBB.1.16 do not provide evidence of its exceptional danger or high expansion capability. Detected differences with previous lineages are probably due to genetic drift, which allows the virus constant adaptability to the host, but they are not necessarily connected to a greater danger. Nevertheless, continuous genome-based monitoring is essential for a better understanding of its descendants and other lineages.

## 1. Introduction

Since late 2019, the world has grappled with the COVID-19 pandemic, caused by the SARS-CoV-2 virus, which was first identified during a pneumonia outbreak in Wuhan, China, in December 2019 [1,2]. The virus quickly gained global attention, and on 11 March 2020, the World Health Organization (WHO) declared it a pandemic when the number of confirmed cases reached 149,295 [3]. As of 16 July 2023, the WHO has reported over 768 million confirmed cases, and over 6.9 million deaths have been reported globally [4]. Although the WHO has recently declared the end of the pandemic emergency, caution is still necessary as SARS-CoV-2 continues to circulate and evolve, and infections remain a significant concern in most countries. Indeed, the WHO emphasizes the need to avoid complacency, as the virus is still evolving and circulating among the global population. Therefore, infections caused by SARS-CoV-2 are expected to persist as a significant problem in many countries [5]. SARS-CoV-2 is an RNA virus with a single-stranded, positive-sense genome. It has a high error rate during RNA replication, leading to mutations that can affect its susceptibility to neutralizing antibodies generated through infection or vaccination, as well as its transmissibility [6]. Throughout the course of the pandemic, SARS-CoV-2 has undergone various changes in its genomic composition, resulting in the emergence of new variants with different rates of transmissibility and dangerousness [5]. The most recent lineage is the SARS-CoV-2 XBB.1.16 variant (nicknamed *Arcturus*), which was first reported on 9 January 2023 and later categorized as a Variant Under Monitoring (VUM) on 22 March 2023 [7]. Subsequently, on 17 April 2023, the XBB.1.16 variant was designated a Variant of Interest (VOI) [7,8]. In epidemiological week 24 (from June 12 to 18), it has become globally dominant [4], surpassing, in terms of prevalence, the other VOI, the XBB.1.5 SARS-CoV-2 variant [4]. Indeed, currently, the SARS-CoV-2 XBB.1.16 variant has been detected in 98 countries, and its genome prevalence has increased by 21.2% compared to the previous epidemiological week, with a genome worldwide prevalence of about 23% [9]. It is very common in Asia, with a genome prevalence of 65%, while in North America, Europe, and Africa, it presents a prevalence of 22%, 12%, and 2%, respectively [4]. Indeed, in Africa, the XBB.1.5 variant is currently still dominant [4].

It is a descendant lineage of the SARS-CoV-2 XBB (nicknamed *Gryphon*), a recombinant of two BA.2 sub-lineages [10]. In addition to the characteristic mutations of its direct ancestor SARS-CoV-2 XBB.1 (nicknamed *Hippogryph*), XBB.1.16 presents three new spike mutations: E180V, K478R, and F486P. The spike profile is very similar to the variant XBB.1.5 [11], and as of now, they present a comparable global risk assessment [4,12]. In addition, like the XBB.1.5 lineage, XBB.1.16 exhibits the same mutations of interest in the spike protein sequence as its ancestor XBB, namely K417N, S477N, N501Y, and P681H [13]. As it often occurs during the early stages of the evolutionary development of a newly formed lineage, numerous concerns emerged initially, primarily relating to its presumed high level of immunoevasion abilities [14]. The genetic characteristics of XBB.1.16 suggest that this new lineage may contribute to an increased number of confirmed cases worldwide. Although there is currently no evidence of heightened severity, there is a moderate indication of an elevated risk of transmission and potential evasion of the immune system. The main concerning feature (shared with its relative lineage XBB.1.5) is given by the variation at position 486, where both XBB.1.5 and XBB.1.16 have F486P instead of the phenylalanine (F) residue found in XBB, which is replaced by a serine (S). Indeed, the F486P substitution represents the primary concern because it is relatively rare when compared to other lineages and the original Wuhan-1 wild type [15]. The role of this mutation is still unknown, but it is possible, like many new mutations, that it grants the virus an enhanced ability to evade the immune system compared to other lineages. Consequently, the World Health Organization (WHO) recommends prioritizing research efforts aimed at better understanding this lineage.

In such a context, the present research involves a comprehensive approach that combines genetic variability/phylodynamic analyses with structural and immunoinformatic studies to gain a thorough understanding of the molecular aspects underlying the epidemiological expansion of SARS-CoV-2 XBB.1.16. The main purpose of this study is to facilitate continuous monitoring of this lineage in order to be prepared in case of a new wave (if any).

## 2. Results

**Figure 1:**
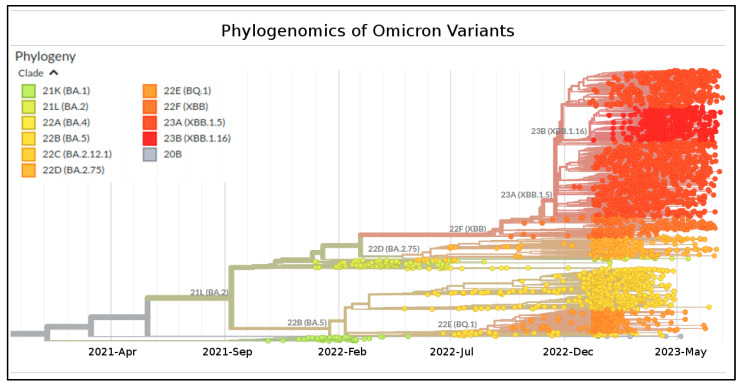
Highlight of the Omicron strains in the time-scaled phylogenetic tree of a representative global subsample of 3126 SARS-CoV-2 genomes sampled between October 2021 and July 2023. Phylogeny has been reconstructed by using nextstrain/ncov (https://github.com/nextstrain/ncov, accessed on 17 July 2023), available at https://gisaid.org/phylodynamics/global/nextstrain/ (accessed on 17 July 2023). The figure has been edited by using the software GIMP 2.8 (available at https://www.gimp.org/downloads/oldstable/, accessed on 17 July 2023). See Table 1 for details on the Nextstrain clade, Pango lineage, and WHO labels.

**Figure 2:**
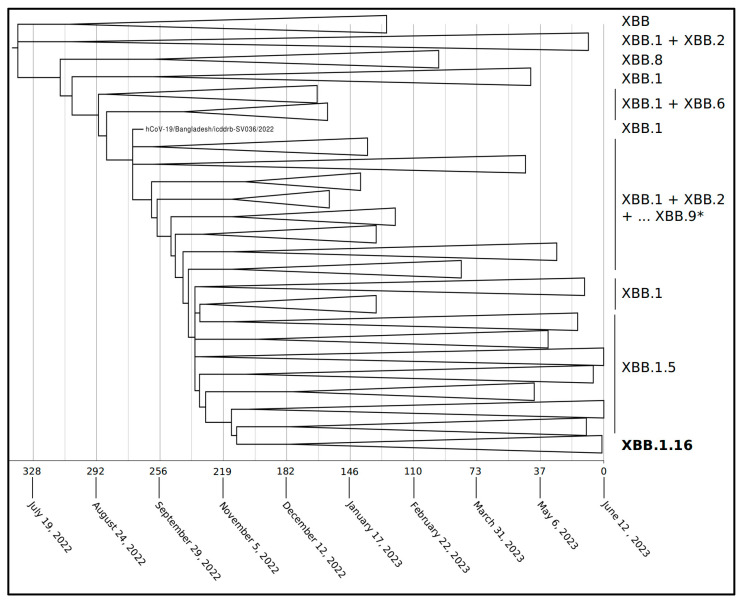
Maximum Clade Credibility tree reconstructed using 1973 whole genomes of XBB and all its descendants downloaded from the GSAID portal (https://gisaid.org/), accessed on 21 June 2023. See File S1 for details on the genomes included in the analyses. All displayed nodes in the tree are well-supported. Values of posterior probabilities for all nodes are between 0.95 and 1 (PP ≥ 0.95). The bar under the tree indicates the time scale expressed in days before 12 June 2023, which represents the most recent sampling date included in the analyzed dataset. * The group of collapsed clade includes all descendants of XBB.

Phylogenomic reconstruction (Figure 1) reveals that the genomes of XBB.1.16 clustered together, forming a monophyletic group (GSAID Clade 23B) within the non-monophyletic clade composed of genomes of its progenitor SARS-CoV-2 XBB (GSAID Clade 22F) and all descendants. See Table 1 for details on the Nextstrain clade, the Pango lineage, and WHO labels. The Bayes Factor analysis conducted on a dataset of 1973 genomes indicates that the Coalescent Bayesian Skyline Model, under the lognormal uncorrelated relaxed clock model, provides a significantly better fit to the data compared to other demographic and clock models tested (2lnBF = 28.1). The Maximum Clade Credibility tree (Figure 2) indicates that all the genomes of XBB.1.16 belong to a clade whose common ancestor is temporally placed around 7 January 2023, with a confidence interval ranging between 28 December 2022 and 12 January 2023.

**Figure 3:**
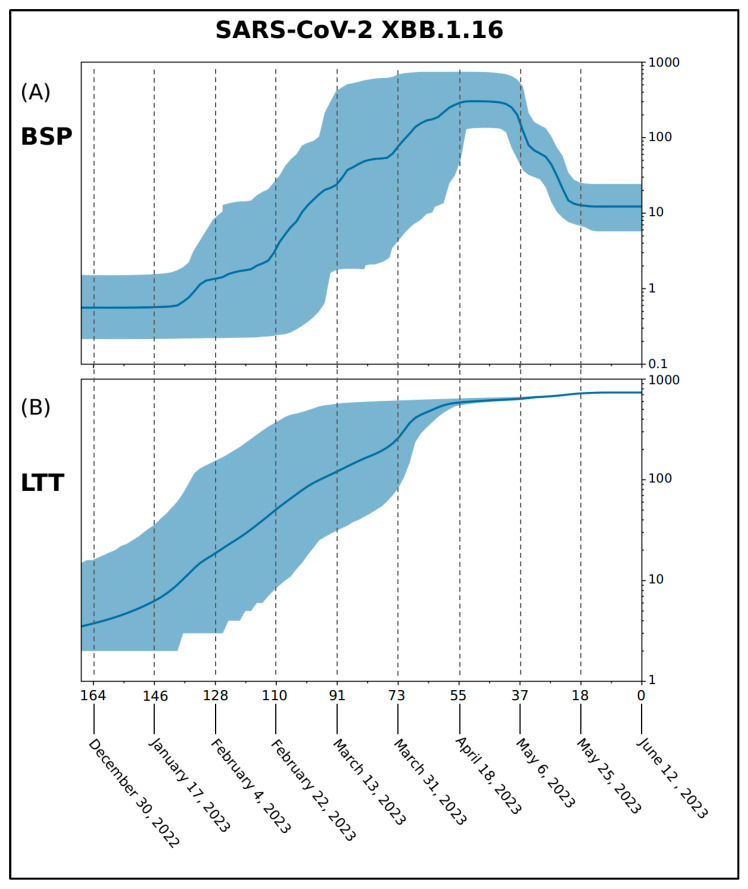
Demographic expansion of the SARS-CoV-2 XBB.1.16 variant is shown as a BSP—Bayesian Skyline Plot (**A**) and LTT—Lineages-Through-Time (**B**). The viral effective population size (*y*-axis) is shown as a function of days (*x*-axis). The solid area represents the 95% high posterior density (HPD) region.

The Bayesian Skyline Plot (BSP) (Figure 3A) demonstrates that the viral population experienced a period of expansion starting about 146–128 days before 12 June 2023 (i.e., between 17 January and 4 February 2023), reaching its peak approximately 55 days before 12 June 2023 (i.e., 18 April 2023). After that, the plateau lasted for less than 20 days, and about 37 days before 12 June 2023 (i.e., 6 May 2023), the viral population size decreased, along with a reduction in genetic variability. The minimum value was reached about 18 days before 12 June 2023 (i.e., 25 May 2023), when the last plateau began and has been ongoing until now. The Lineages-Through-Time plot (LTT) (Figure 3B) suggests that the increase in the number of lineages began around late 2022. The growth continues with few fluctuations until 55 days before 12 June 2023 (i.e., 18 April 2023). After that, the number of lineages stopped growing. The evolutionary rate, estimated using the Coalescent Bayesian Skyline Model (under the lognormal uncorrelated relaxed clock model), amounts to approximately 3.95 × 10^−4^ [95% HPD 2.38 × 10^−5^–7.37 × 10^−4^] subs/sites/years.

**Figure 4:**
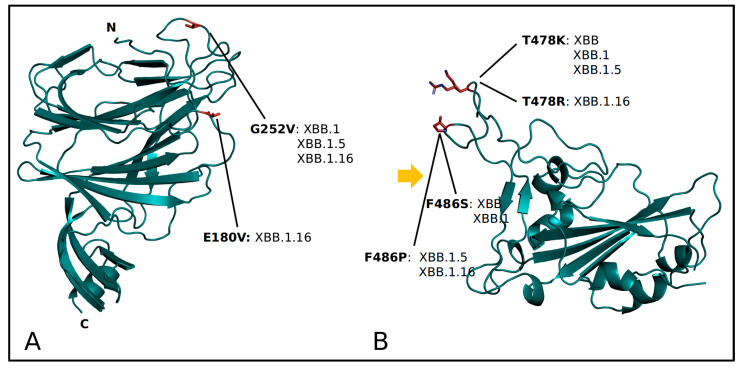
Ribbon model of SARS-CoV-2 NTD (**A**) and RBD (**B**). Mutations characterizing the four variants are labeled, indicating the original residue, the sequence position, and the replacing residue, along with associated variants. In (**A**), N and C mark the N- and the C-terminus, respectively. In (**B**), the orange arrow marks the interface to ACE2.

The structural evolution of the SARS-CoV-2 spike from the original strain to Omicron involves fine-tuning the spike’s physico-chemical properties [16]. In this work, the structural properties of four related variants have been compared. Characterizing mutations of each variant of RBD and NTD are reported in Table S1 and Figure 4.

The net charge has been calculated with PROPKA for each of the four NTDs and RBDs as a quantitative measure of differences among the surface electrostatic surfaces (Table 2). The comparison suggests that the RBD has reached a charge plateau corresponding to a value of about +5.40, irrespective of the specific variant considered. The distribution of the surface potential is also similar in the considered variants (Figure S1). The Wilcoxon test confirmed that there is no significant difference among the RBD net charges. At variance with the RBD, the NTD shows charge variability. Indeed, it appears clearly negative in XBB, XBB.1, and XBB.1.5, while it is close to neutrality in XBB.1.16, as expected by the substitution of Glu180 with the neutral Val. Accordingly, the distribution of the surface potential is influenced by this substitution (Figure S2). In this case, the Wilcoxon test indicates that the XBB.1.16 NTD has a significantly higher charge with respect to the other variants (*p*-value ≈ 10^−16^).

The mutations’ effect at the ACE2-RBD interface of the four variants has been assessed by the prediction of interaction energy by three independent methods: FoldX5, PRODIGY, and MM/GBSA (Table 3).

**Figure 5:**
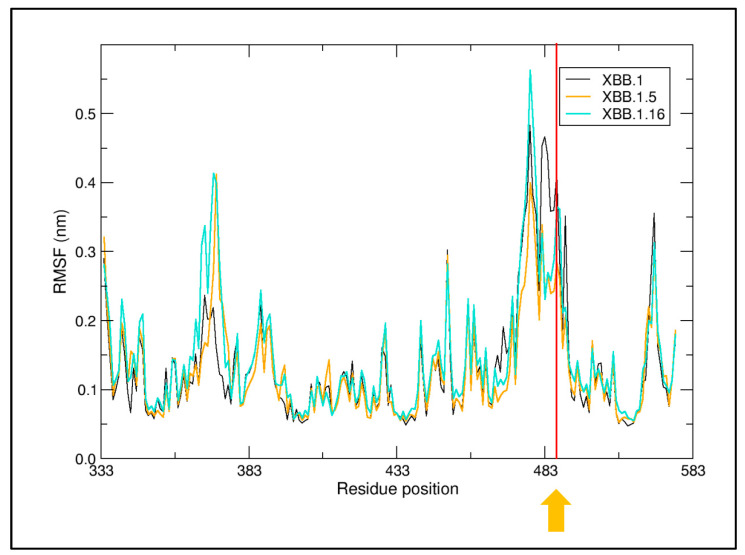
Root-Mean-Square Fluctuation (RMSF) of XBB.1, XBB.1.5, and XBB.1.16 RBDs. The displayed RMSF is an average for each residue. The orange arrow and the red line mark approximately the sequence position 486.

Within the limits of their accuracy, the results of the three methods indicate that the affinity for ACE2 is similar in the four variants. However, slightly higher stability is attributed to XBB.1.5 and XBB.1.16 by FoldX 5.0 and MM/GBSA analysis. The Wilcoxon test indicates that the energy differences calculated by Foldx5 are statistically significant (*p*-value < 0.02). Interestingly, the two variants possess the ACE2 interface mutation F486P instead of F486S of XBB and XBB.1. The same mutations have been detected also in XBF [17]. As already reported [11], the replacement of Phe with Pro partly disrupts the hydrophobic interaction with the ACE2 residues L61, M64, and Y65. Moreover, a comparison of the Root-Mean-Square Fluctuation in XBB.1, XBB.1.5 and XBB.1.16 show that the P486 decreases RBD local flexibility at the ACE2 interface (Figure 5). Apparently, XBB.1.16 R486 promotes a moderate increase in local flexibility (Figure 5). It should also be noted that XBB.1.5 and XBB.1.16 display increased local flexibility around position 370 with respect to XBB.1. As XBB and XBB.1 RBDs share the same sequence, only XBB.1 has been considered in this analysis. The molecular dynamics of XBB.1 and XBB.1.16 NTDs suggest that the residue substitutions do not significantly alter the local chain flexibility as measured by the RMSF plots (Figure S3).

## 3. Discussion

The SARS-CoV-2 Omicron XBB.1.16 variant is one of the most recently discovered lineages. Like any newly identified variants, XBB.1.16 needs a rigorous assessment of its genomic variances in comparison to previous lineages. This evaluation is essential to determine its potential for spreading, contagiousness, and pathogenicity aspects, including its ability to evade the immune system, also considering that it has been classified as a Variant of Interest (VOI) [7,8]. In this research, our primary objective was to achieve a comprehensive comprehension of the evolutionary and structural characteristics of the SARS-CoV-2 XBB.1.16 variant. To achieve this purpose, we conducted an analysis that encompassed all the accessible genomes available in the GSAID database as of 10 July 2023. Phylogenomic reconstruction indicated that the genomes of XBB.1.16 (GSAID Clade 23B) clustered together within the broader cluster of GSAID Clade 22F (XBB.1.16) and all its descendants. This is not surprising since XBB.1.16 is also a descendant of XBB. One of the most significant findings is related to the evolutionary condition of XBB.1.16, which appears as an evolutionary dead end with no further epidemiologically relevant descendants. If this evolutionary condition is confirmed, it would indicate an accumulation of neutral loss-of-function mutations in XBB.1.16 and its descendant lineages soon. Typically, such lineages are characterized by numerous nucleotide mutations but few or no amino acid mutations in important genes. Furthermore, the branch lengths in the phylogenetic tree indicate a lack of rapid diversification, which is typical of a dangerous lineage in the early stages of its evolutionary path. Recent variants and lineages that emerged in 2022 and 2023 displayed similar conditions, which raised significant concerns. These variants included BA.2.75, BQ.1, XBB, XBB.1.5, BF.7, CH.1.1, and XBF. Initially, there were worries about their potential impact, but further molecular analysis revealed that they had limited demographic expansion capability and low chances of becoming dangerous [10,11,17,18,19,20,21]. In the past, a similar situation was observed with the BA.2.12.1 variant, which did not give rise to further sub-lineages and gradually decreased in prevalence until it almost disappeared from the genomic global sequence [9]. The phylodynamic reconstruction, conducted using a dataset consisting of 1973 genomes, revealed that among all the lineages included (comprising XBB and all its descendant sub-lineages), only XBB.1.16 displayed a monophyletic condition. In other words, XBB.1.16 formed a distinct and unique cluster in the evolutionary tree, closely related to but clearly distinct from other lineages in the dataset. In the Maximum Clade Credibility tree, the common ancestor of all XBB.1.16 genomes is temporally placed around 7 January 2023 (ranging between 28 December 2022 and 12 January 2023). This date is fully consistent with the earliest documented samples that are dated back to 9 January 2023 [8]. It is interesting to note that XBB.1.16 was designated a Variant of Interest (VOI) only on 17 April 2023 due to its documented reports in 98 countries and became dominant in the second half of June [4]. This is not characteristic of a dangerous variant, which typically spreads rapidly and affects a large population. Indeed, it showed slow growth simultaneously with the reduction in the prevalence of XBB.1.5 [11]. The BSP analysis, based on 1229 complete XBB.1.16 genomes collected between 4 January 2023 and 12 June 2023, indicates a relatively low level of genetic variability. Indeed, after an initial period of low and stable genetic variability, in late January the increase in genetic variability and the number of lineages started. Accordingly, during the same period, the first increase in the viral population size was observed. During its mild growth, which lasted about 90 days, the viral population size experienced several slight fluctuations (without any significant changes) and peaked on 18 April 2023. Since then, the first plateau began, lasting around 20 days. In early May, the genetic variability started decreasing, and by late May, the viral population size was similar to the levels observed in early March. Since then, the last plateau began and has persisted until now, with both genetic variability and viral population size showing no further fluctuations. The LTT analysis indicates that the increase in lineages was mild, too, and the maximum number was reached a few days before the viral population size peaked. The current size appears to be stable, lacking significant changes for almost one month. This is not the typical trend of a lineage that is about to experience a rapid expansion in terms of population size and contagiousness, which was observed during the early stages of the pandemic when variability surged rapidly in a steep curve. On the contrary, the observed trend is similar to that of several previously mentioned recent variants (i.e., BA.2.75, BQ.1, XBB, XBB.1.5, BF.7, CH.1.1, and XBF), which initially raised concerns but, after comprehensive genome-based analysis, showed no evidence of them being particularly dangerous or having a high expansion capacity [10,11,17,18,19,20,21]. The situation of XBB.1.16 aligns with a scenario typical of an evolutionary lineage that exhibits new characteristics compared to its direct ancestor (XBB.1), but as of now, these new features do not contribute to an abnormal expansion. Moreover, the absence of lineage growth over time provides additional evidence for the lack of an increase in the number of distinct genetic variants in recent periods. The estimated evolutionary rate for XBB.1.16 is 3.95 × 10^−4^ subs/site/year, with a narrow range of 2.38 × 10^−5^–7.37 × 10^−4^ subs/sites/years. This further confirms the low level of genetic variation and limited potential for demographic expansion. It is slower than the previous dominant variant, XBB.1.5, which presented a mutation rate of 6.9 × 10^−4^ subs/sites/years [11]. In addition, it is interesting to note that it is slightly lower than its direct progenitor (XBB.1), which presented 6.3 × 10^−4^ subs/sites/years [10] as the evolutionary rate. This last piece of information represents an evolution that always progresses forward and never backward, generating lineages that are even slower and less dangerous due to genetic drift. Indeed, if XBB.1.16 were a highly contagious and dangerous variant, its evolutionary rate would be expected to be higher. For instance, the evolutionary rate of the initial SARS-CoV-2 lineage during the early stages of the pandemic was approximately 6.58 × 10^−3^ subs/sites/years [22], which means that the new variant (along with the previous recent variants) presents a 10^−1^ slower rate of evolution compared to the Wuhan-Hu-1 variant.

Comparison among the four variants suggests that the RBD, the essential player in the spike cell infection process, has reached a net charge plateau at about +5.4 since the Omicron variant emerged. Apparently, as far as we know now, this value represents an upper limit to the RBD charge, possibly related to the thermodynamic stability of the domain. Interestingly, the NTD has a more fluctuating net charge in different variants that may reflect its different functional role [23]. The theoretical affinity for ACE2 does not seem to change among the variants considered despite a decrease of local flexibility in XBB.1.16 and XBB.1.5 caused by the presence of Pro in position 486. Indeed, this substitution causes an increase in local chain flexibility. It should be noted that the same substitution has been detected in other variants, such as XBF [17]. Substitution of K478 with Arg in XBB.1.16 does not appear to significantly alter the local flexibility. The XBB.1.16 NTD displays an increased overall net charge due to the elimination of the negatively charged Glu180 present in the wild-type. However, this substitution does not appear to induce significant changes in local chain flexibility. Overall, the XBB.1.16 variant does not possess structural characteristics manifestly different from those of the parent lineages. However, it cannot be excluded that fine-tuning of structural properties, such as surface charge distribution or local flexibility, may influence spike interaction with host molecular components, for example, the immune system, thereby modulating virus behavior.

As per evolutionary theories, the new SARS-CoV-2 variants frequently demonstrate the ability to escape antibodies, but this does not necessarily mean they have a higher transmission capability or increased virus pathogenicity. Based on the available data, it seems that SARS-CoV-2 XBB.1.16 is a novel variant without heightened infectivity or pathogenicity in comparison to its direct ancestor XBB.1 and the previously dominant variant XBB.1.5. XBB.1.16 does not present an actual worldwide threat; rather, it comprises recently evolved lineages that have acquired specific characteristics through genetic drift. While these features might be theoretically associated with enhanced fitness, they do not actually confer a substantial advantage in terms of fitness. In theory, the emergence of new viral variants poses a potential challenge as these variants can accumulate genetic changes through drift, potentially enabling them to evade pre-existing antibodies. This genetic variability allows the virus to explore different genetic pathways, potentially leading to altered antigenic properties. Despite this, current treatments and preventive measures still hold promise. Many treatments target multiple components of the virus or the host-virus interaction, reducing the risk of complete ineffectiveness due to a single genetic change. Furthermore, advancements in vaccine development and therapeutic strategies have shown adaptability in the face of evolving variants. Continuous monitoring of viral evolution coupled with swift adjustments to medical interventions ensures that while the virus may navigate the complex landscape of genetic diversity, our scientific approach remains resilient and capable of responding effectively.

## 4. Materials and Methods

### 4.1. Phylodynamic Analyses

The initial genomic assessment of the SARS-CoV-2 XBB.1.16 Omicron variant (GSAID Clade 23B) was conducted using a globally focused subsampling approach over the past six months. The analysis utilized the nextstrain/ncov tool (https://github.com/nextstrain/ncov, accessed on 17 July 2023), which is available at https://gisaid.org/phylodynamics/global/nextstrain/ (accessed on 17 July 2023) and includes all the genomes belonging to the Omicron variants. The resulting phylogenetic tree was edited using GIMP 2.8 software (available at https://www.gimp.org/downloads/oldstable/, accessed on 17 July 2023).

Following the initial assessment, a subset of 1973 genomes of XBB.1.16, XBB.1.5, and XBB, and all descendants (different than XBB.1.16 and XBB.1.5) were selected for further, more focused phylogenetic analyses. The genomes were aligned using the L-INS-I algorithm implemented in the software Mafft 7.471 [24]. Subsequent manual editing of the alignments was conducted using Unipro UGENE software v.35 [25]. To identify the most suitable probabilistic model of genome evolution, we employed the software jModeltest 2.1.1 [26] by performing a maximum likelihood optimized search. For investigating the phylogenomic relationships among variants and their divergence times, we employed Bayesian Inference (BI) using the software BEAST 1.10.4 [27]. The BI analysis ran for 200 million generations, employing various demographic and clock models. To select the best representative output, the Bayes Factor test [28] was utilized, where we compared the 2lnBF values of the marginal likelihoods, by using the software Tracer 1.7 [29]. The final runs have been performed using the Coalescent Bayesian Skyline Model under the lognormal uncorrelated relaxed clock model. A time-based phylogenetic tree was drawn and visualized by using TreeAnnotator (Beast package) and FigTree 1.4.0 (available at http://tree.bio.ed.ac.uk/software/figtree/, accessed on 26 July 2023).

BEAST software (version 1.10.4) was utilized to estimate the evolutionary rate, the Bayesian Skyline Plot (BSP), and Lineages-Through-Time (LTT) for the XBB.1.6 variant. For this analysis, a subset of 1299 genomes was selected, and the analysis ran for 300 million generations using the Bayesian Skyline Model with the uncorrelated log-normal relaxed clock model.

All datasets used in this study were constructed by downloading the genomes from the GISAID portal (https://gisaid.org/) accessed on 10 July 2023. For more comprehensive information about the genomes included in the dataset and authorship, please refer to File S1.

### 4.2. Structural Analyses

Homology models of the RBDs (receptor-binding domain) and NTDs (N-terminal domain) for XBB, XBB.1, XBB.1.5, and XBB.1.16 were generated using Modeller 10.3 [30]. The templates used for homology modeling were taken from the PDB codes 6M0J (for RBD) and 7B62 (for NTD). Model structures were displayed and examined by using the graphical software PyMOL [31]. Side-chain conformations of the homology models were optimized using the FoldX 5.0 program with the “RepairPDB” function [32]. Fluctuations of side-chain conformations and interactions were studied by generating 100 alternative homology models for the RBD and the NTD domains. FoldX 5.0 was also used to optimize side-chain conformations, energy, and non-bonding interactions for each of the 100 models. To evaluate their average and standard error, structural properties were calculated across all models. PROPKA3 [33] was used to predict domain net charges at pH 7.0. The surface electrostatic potential was calculated using the APBS method [34], and SURFMAP software [35] was employed to display a two-dimensional map representing the distribution of physicochemical features over protein surfaces. This method of “molecular cartography” allows for the analysis and comparison of different features. Furthermore, complex models between ACE2 and the RBDs of the variants XBB/XBB.1, XBB.1.5, and XBB.1.16 were constructed using Modeler and the template from the PDB structure 6M0J. Interaction energies between the spike RBDs and ACE2 were predicted using “AnalyseComplex” from the Foldx 5.0 suite, as well as MM/GBSA (Molecular Mechanics/Generalized Born Surface Area) available in HawkDock server [36], and PRODIGY [37]. Foldx 5.0 employs an empirical force field that accounts for various free energy terms, including electrostatic interactions, hydrogen bonds, desolvation, and van der Waals contacts. MM/GBSA HawkDock calculates binding free energies by combining molecular mechanics calculations and continuum solvation methods. PRODIGY predicts binding affinities through scrutiny of subunit interface contacts.

The molecular dynamics simulations were conducted using GROMACS 2022.5 [38] with the AMBER99SB-ILDN force field [39]. The RBD structure was solvated in a dodecahedral box with TIP3P water molecules, and a 1.5 nm distance was kept to the box edge. The system was neutralized to a final concentration of 0.15 M NaCl. All simulations were performed in periodic boundary conditions. After minimization, the system underwent 100 ps of NVT and NPT equilibration at 300 K. The production simulation was run for 100 ns with a 2 fs time-step. Trajectories were visualized using VMD 1.9.3 [40], and analysis was performed with GROMACS ttools and the XMGRACE software package Version 5.1.19 [41]. Root- Mean-Square Fluctuation was calculated as an average for each residue over the 100 ns production simulation.

The statistical tests have been carried out within the R-studio environment (RStudio Team (2020). RStudio: Integrated Development for R. RStudio, PBC, Boston, MA URL http://www.rstudio.com/, accessed on 27 July 2023). The significance of observed differences in distribution medians has been tested with the two-sample Wilcoxon test applied systematically to compare all variant pairs [42].

## 5. Conclusions

In conclusion, the genetic and structural analysis of the SARS-CoV-2 XBB.1.16 variant suggests that, despite harboring several spike mutations of interest, there is currently no evidence to indicate exceptional danger or high expansion capability. Despite being the current dominant variant, reported in 98 countries [4], the expansion of XBB.1.16 seems to be even slower than that of the previous dominant variant, XBB.1.5. Based on current data, there is no immediate cause for alarm. The viral population size reached its peak on 18 April 2023, and has been decreasing since early May, which is far from the epidemiologically dangerous lineage observed at the beginning of the pandemic, where the population size exhibited an extremely steep curve. Due to this rapid evolution, primarily driven by natural selection, the emergence of new mutations is not a novel or occasional occurrence but an assured phenomenon that periodically repeats. Accordingly, throughout the pandemic, SARS-CoV-2 has experienced a significant number of mutations, leading to the emergence of numerous lineages and sub-lineages with different expansion capabilities [5]. It is normal for new variants to arise as part of the evolutionary process. However, it is crucial to maintain vigilance against the pandemic and the potential generation of further variants. Continuous genome-based surveillance is essential for better understanding this phenomenon. Continuous monitoring of XBB.1.16 and its descendants, along with all other lineages, is necessary to identify and predict significant changes in the genomic composition and diffusion capability in order to predict new further waves (if any).

## Figures and Tables

**Table 1:** Nextstrain Clade, Pango Lineage, and WHO labels of the investigated lineages are shown in Figure 1.

Nextstrain Clade	Pango Lineage	WHO Label
21K (Omicron)	BA.1	o (Omicron)
21L (Omicron)	BA.2	o (Omicron)
22C (Omicron)	BA.2.12.1	o (Omicron)
22D (Omicron)	BA.2.75	o (Omicron)
22A (Omicron)	BA.4	o (Omicron)
22B (Omicron)	BA.5	o (Omicron)
22E (Omicron)	BQ.1	o (Omicron)
22F (Omicron)	XBB	o (Omicron)
23A (Omicron)	XBB.1.5	o (Omicron)
23B (Omicron)	XBB.1.16	o (Omicron)

**Table 2:** Net charge comparison for NDT and RBD among lineages.

	XBB	XBB.1	XBB.1.5	XBB.1.16
NTD	−1.24 ± 0.00	−1.18 ± 0.01	−1.18 ± 0.01	−0.06 ± 0.03
RBD	5.45 ± 0.02	5.45 ± 0.02	5.42 ± 0.01	5.57 ± 0.02

**Table 3:** Predicted interaction energy between ACE2 and variant RBDs expressed in Kcal/mol.

	XBB	XBB.1	XBB.1.5	XBB.1.16
FoldX_5.0	−3.54 ± 0.30	−3.54 ± 0.30	−4.57 ± 0.27	−4.12 ± 0.33
PRODIGY	−11.48 ± 0.05	−11.48 ± 0.05	−10.84 ± 0.05	−10.94 ± 0.04
MM/GBSA	−60.82 ± 0.99	−60.82 ± 0.99	−62.44 ± 2.28	−62.49 ± 0.68

## Data Availability

Genomes analyzed in the present study were taken from the GSAID database and are available at https://gisaid.org/ (accessed on 21 June 2023).

## Supplementary Materials

The supporting information can be downloaded at: https://www.mdpi.com/article/10.3390/ijms241713573/s1.
