# Supplementary material for: Integrative Genome-Based Survey of the SARS-CoV-2 Omicron XBB.1.16 Variant
Source: Int J Mol Sci. 2023 Sep 1;24(17):13573. doi: 10.3390/ijms241713573 (PMC10487968; doi:10.3390/ijms241713573)
Supplement: Supplementary file 1 [file ijms-24-13573-s001.zip › Figures.pdf]

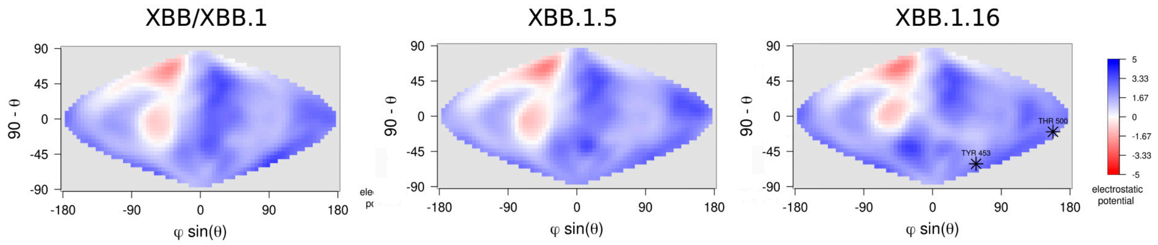

Figure S1: The electrostatic potential surface of four RBD domains was projected onto a two-dimensional map. The color scale for the XBB.1.16 map is provided alongside. The electrostatic potential values are expressed in kT/e units. The map axes show the projected polar coordinates of the domains. Two asterisks indicate the positions of residues T453 and T500, which are part of the interface with ACE2.

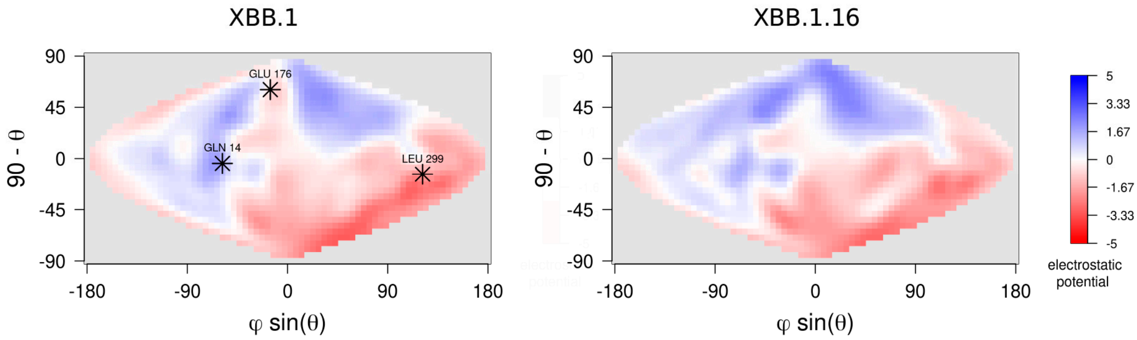

Figure S2: Projection on a two-dimensional map of the electrostatic potential surface of NTDs of XBB1 and XBB.1.16 domains. Color scale and axis are as in Figure S1. Asterisks indicate the position of the N- (Gln14) and C-terminal (Leu299) residues and the mutated site Glu180 (indicated as 176).

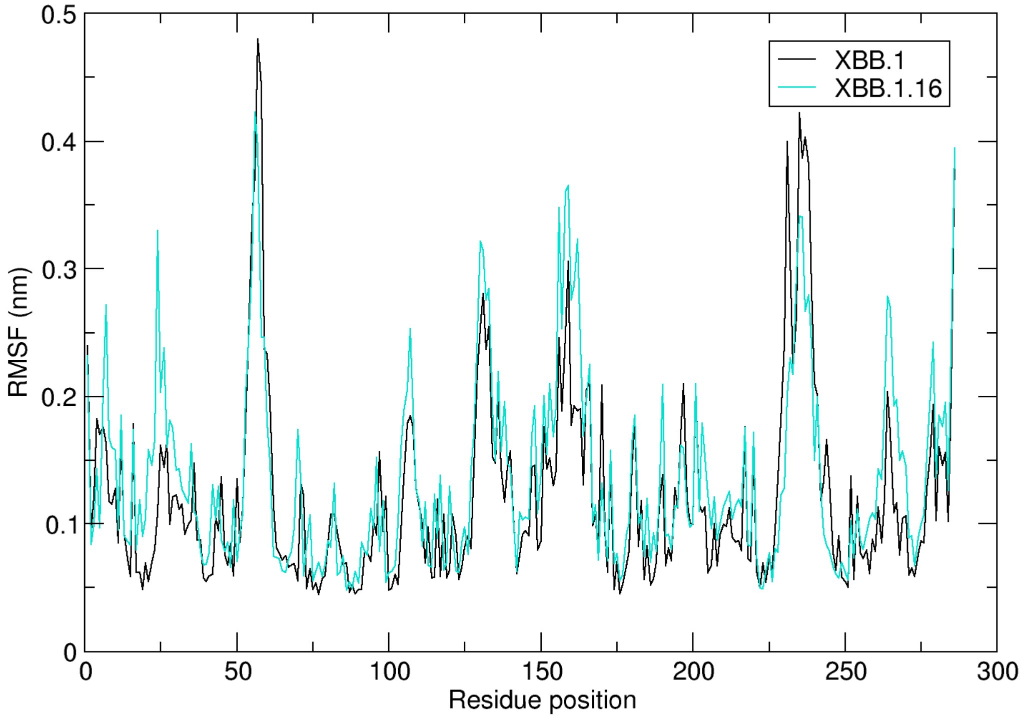

Figure S3: Root Mean Square Fluctuation (RMSF) of XBB.1, and XBB.1.16 NYDs. The displayed RMSF is an average for each residue.
