# Supplementary material for: Integrative Genome-Based Survey of the SARS-CoV-2 Omicron XBB.1.16 Variant
Source: Int J Mol Sci. 2023 Sep 1;24(17):13573. doi: 10.3390/ijms241713573 (PMC10487968; doi:10.3390/ijms241713573)
Supplement: Supplementary file 1 [file ijms-24-13573-s001.zip › File_S1 Acknowledgement and reference of Authors who deposited analyzed genomes in GSAID portal.pdf]

SUPPLEMENTAL TABLE

Data Availability

GISAID Identifier:	EPI_SET_230621vu
doi:	10.55876/gis8.230621vu

All genome sequences and associated metadata in this dataset are published in GISAID’s EpiCoV database. To view the contributors of each individual sequence with details such as accession number, Virus name, Collection date, Originating Lab and Submitting Lab and the list of Authors, visit 10.55876/gis8.230621vu

Data Snapshot

- EPI_SET_230621vu is composed of 1,299 individual genome sequences.
- The collection dates range from 2023-01-04 to 2023-06-12;
- Data were collected in 27 countries and territories;
- All sequences in this dataset are compared relative to hCoV-19/Wuhan/WIV04/2019 (WIV04), the official reference sequence employed by GISAID (EPI_ISL_402124). Learn more at https://gisaid.org/WIV04.
