# Supplementary material for: Integrative Genome-Based Survey of the SARS-CoV-2 Omicron XBB.1.16 Variant
Source: Int J Mol Sci. 2023 Sep 1;24(17):13573. doi: 10.3390/ijms241713573 (PMC10487968; doi:10.3390/ijms241713573)
Supplement: Supplementary file 1 [file ijms-24-13573-s001.zip › Table_S1.pdf]

Table S1: Comparison of the XBB, XBB.1, XBB.1.5 and XBB.1.16 mutations in the NTD and RBD regions of the Spike. In red bold font are listed the mutations of interest.

XBB	XBB.1	XBB.1.5	XBB.1.16
T19I	T19I	T19I	T19I
L24S	L24S	L24S	L24S
P25-	P25-	P25-	P25-
P26-	P26-	P26-	P26-
A27-	A27-	A27-	A27-
V83A	V83A	V83A	V83A
G142D	G142D	G142D	G142D
Y144-	Y144-	Y144-	Y144-
H146Q	H146Q	H146Q	H146Q
			E180V
Q183E	Q183E	Q183E	Q183E
V213E	V213E	V213E	V213E
G252V	G252V	G252V	G252V
G339H	G339H	G339H	G339H
R346T	R346T	R346T	R346T
L368I	L368I	L368I	L368I
S371F	S371F	S371F	S371F
S373P	S373P	S373P	S373P
S375F	S375F	S375F	S375F
T376A	T376A	T376A	T376A
D405N	D405N	D405N	D405N
R408S	R408S	R408S	R408S
K417N	K417N	K417N	K417N
N440K	N440K	N440K	N440K
V445P	V445P	V445P	V445P
G446S	G446S	G446S	G446S
N460K	N460K	N460K	N460K
S477N	S477N	S477N	S477N
T478K	T478K	T478K	
			T478R
E484A	E484A	E484A	E484A
F486P		F486P	F486P
F490S	F490S	F490S	F490S
Q498R	Q498R	Q498R	Q498R
N501Y	N501Y	N501Y	N501Y
Y505H	Y505H	Y505H	Y505H
D614G	D614G	D614G	D614G
H655Y	H655Y	H655Y	H655Y
N679K	N679K	N679K	N679K
P681H	P681H	P681H	P681H
N764K	N764K	N764K	N764K
D796Y	D796Y	D796Y	D796Y
Q954H	Q954H	Q954H	Q954H
N969K	N969K	N969K	N969K
